# Incorporation of Amino Acids with Long-Chain Terminal Olefins into Proteins

**DOI:** 10.3390/molecules21030287

**Published:** 2016-02-29

**Authors:** Matthias P. Exner, Sebastian Köhling, Julie Rivollier, Sandrine Gosling, Puneet Srivastava, Zheni I. Palyancheva, Piet Herdewijn, Marie-Pierre Heck, Jörg Rademann, Nediljko Budisa

**Affiliations:** 1 Institute of Chemistry, Technische Universität Berlin, Mueller-Breslau-Strasse 10, 10623 Berlin, Germany; m.p.exner@gmx.net (M.P.E.); zheni.ivanova@gmail.com (Z.I.P.); 2 Institute of Pharmacy, Freie Universität Berlin, Königin-Luise-Str. 2+4, 14195 Berlin, Germany; koehling@zedat.fu-berlin.de (S.K.); joerg.rademann@fu-berlin.de (J.R.); 3 Service de Chimie Bioorganique et de Marquage, iBiTecS, CEA, 91191 Gif-sur-Yvette, France; julie.rivollier@cea.fr (J.R.); sgosling@isthmus.fr (S.G.); marie-pierre.heck@cea.fr (M.-P.H.); 4 Medicinal Chemistry, Rega Institute for Medical Research, KU Leuven, Minderbroedersstraat 10, 3000 Leuven, Belgium; puneet.srivastava@rega.kuleuven.be (P.S.); piet.herdewijn@rega.kuleuven.be (P.H.)

**Keywords:** stop codon suppression, protein decoration, noncanonical amino acid, pyrrolysyl-tRNA synthetase, thiol-ene coupling, metathesis

## Abstract

The increasing need for site-specific protein decorations that mimic natural posttranslational modifications requires access to a variety of noncanonical amino acids with moieties enabling bioorthogonal conjugation chemistry. Here we present the incorporation of long-chain olefinic amino acids into model proteins with rational variants of pyrrolysyl-tRNA synthetase (PylRS). *N*_ε_-heptenoyl lysine was incorporated for the first time using the known promiscuous variant PylRS(Y306A/Y384F), and *N*_ε_-pentenoyl lysine was incorporated in significant yields with the novel variant PylRS(C348A/Y384F). This is the only example of rational modification at position C348 to enlarge the enzyme’s binding pocket. Furthermore, we demonstrate the feasibility of our chosen amino acids in the thiol-ene conjugation reaction with a thiolated polysaccharide.

## 1. Introduction

The emergence of stop codon suppression to incorporate noncanonical amino acids (ncAAs) over the last fifteen years enabled the production of proteins that are site-specifically endowed with various chemical handles, mimics of natural posttranslational modifications (PTMs) and spectroscopic probes [1,2,3,4]. In order to incorporate PTMs synthetically and specifically, the use of versatile moieties that are capable of undergoing orthogonal bioconjugation reactions is appealing, particularly when considering various ncAAs with alkene, alkyne and azide groups are readily available. In this context, the incorporation of alkene-bearing amino acids for the specific labeling and subsequent conjugation reactions is an exceptionally promising pursuit. The olefin moiety exhibits good reactivity and selectivity in several conjugation reactions, many of which can be performed in a protein context, like thiol-ene coupling [5,6], tetrazine ligation [7], tetrazole-click reaction [8], oxidative Heck reaction [9] and even olefin cross-metathesis [10,11,12]. All reactions have been recently reviewed [13]. Olefins are stable and chemically inert outside these specific conditions, and do not easily react with substances and structures found in organisms. Furthermore, being absent from natural protein compositions, olefins are an ideal tool for site-selective and biocompatible ligand attachment onto proteins. For these reasons, olefinic amino acids have been incorporated into proteins as methionine surrogates [14], or site-specifically using orthogonal pairs of aminoacyl-tRNA synthetase and tRNA based on *Escherichia coli* leucyl-tRNA synthetase [10], *Methanocaldococcus jannaschii* tyrosyl-tRNA synthetase [15] and pyrrolysyl-tRNA synthetases from several species [3].

**Figure 1:**
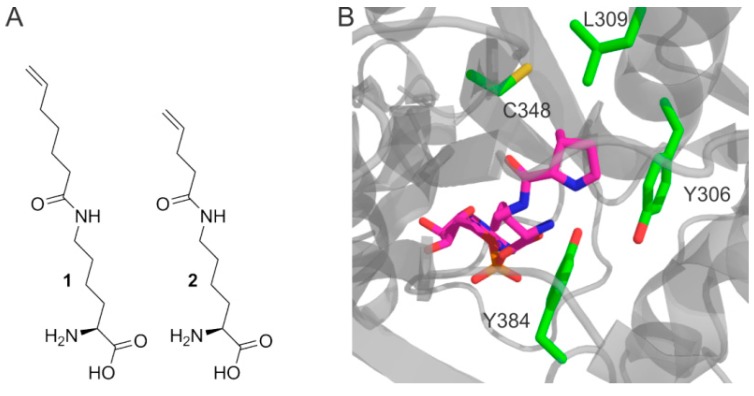
Incorporation of noncanonical amino acids. (**A**) Amino acids *N*_ε_-heptenoyllysine (Hek, **1**) and *N*_ε_-pentenoyllysine (Pek, **2**) used in this study; (**B**) The amino acid binding pocket of *Methanosarcina* *mazei* PylRS binding the natural substrate pyrrolysine-AMP (pink), from PDB id 2ZIM [16]. Residues modified in this study are highlighted (green).

We speculated that *N*_ε_-pentenoyllysine (Pek) and *N*_ε_-heptenoyllysine (Hek) (see Figure 1A) would be promising targets for post-translational chemical modification. The olefinic group is attached to the lysine moiety via an amide bond, exhibiting higher chemical stability than the commonly used carbamate functionality. Furthermore, the double bond is presented at the terminus of a long, flexible chain which serves as a spacer to the protein surface. This should provide good accessibility for chemical protein decoration and reduce the impact of chemical modifications on protein stability and function.

Pek was recently identified as a low-affinity substrate for *Methanosarcina barkeri* pyrrolysyl-tRNA synthetase (*Mb*PylRS) [6]. All PylRS are known to be very promiscuous enzymes, exhibiting little substrate recognition beyond the required ε-amide group, with a hydrophobic pocket which accommodates the head group [16,17]. It has been shown, that enlarging the hydrophobic pocket allows the incorporation of noncanonical amino acids with longer or bulkier head groups [18,19,20]. This inspired us to assess the incorporation of Hek and Pek with variants of *Methanosarcina mazei* PylRS (*Mm*PylRS) with rationally enlarged amino acid binding pockets, as illustrated in Figure 1B.

## 2. Results

**Figure 2:**
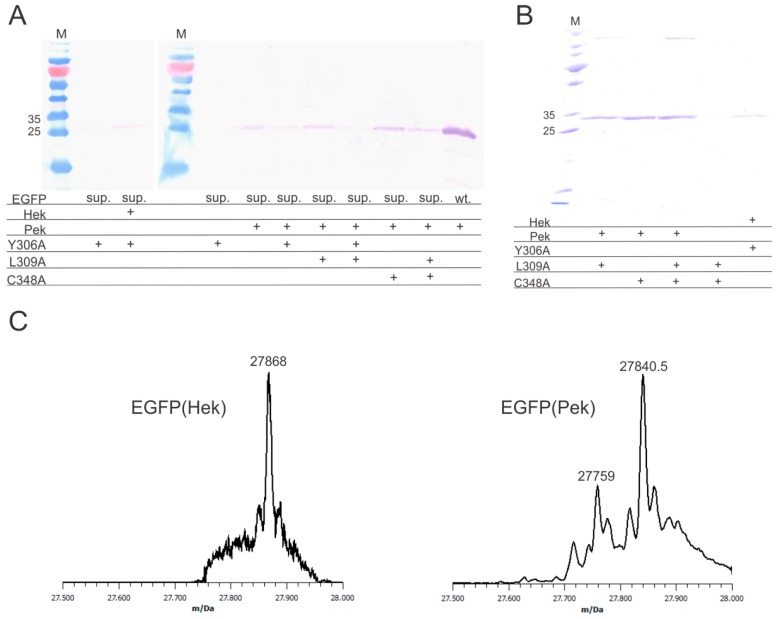
Expression, purification and analysis of the target protein (EGFP)(*N*_ε_-heptenoyllysine (Hek)) and EGFP(*N*_ε_-pentenoyllysine (Pek)). (**A**) Western blot analysis of cell lysates after expression. The same amount of cell lysate was added to each lane for each individual blot. EGFP(N150amber) (sup.)was used for suppression testing, with wild-type EGFP (wt.) as a control. A quantity of 2 mM of either Hek or Pek were added to the cultures. All pyrrolysyl-tRNA synthetase (PylRS) variants used for suppression had the mutation Y384F with additional mutations indicated; the + indicates the presence of an amino acid or mutation; (**B**) EGFP(150Hek) expressed with PylRS(Y306A/Y384F) and EGFP(150Pek) expressed with different variants after purification. The eluates were adjusted to the same volume, and an identical sample volume was applied to each lane; the + indicates the presence of an amino acid or mutation; (**C**) Deconvoluted ESI-MS spectra of EGFP(150Hek) (calculated mass: 27,868.5 Da; found mass: 27,867 Da) and EGFP(150Pek) (calculated mass: 27,840.5 Da; found mass: 27,840.5 Da). An additional peak at 27,759 Da was found, which may correspond to EGFP(150Gln) (calculated mass 27,759 Da).

Using enhanced green fluorescent protein (EGFP) with the internal stop codon N150amber as a model, we initially attempted to incorporate Pek and Hek with *Mm*PylRS(Y384F), a well-known rational mutant which promotes the aminoacylation reaction without significantly altering the specificity [18]. We found that Pek is indeed accepted by the enzyme and EGFP(150Pek) was produced in low yields (less than 1 mg per liter of culture volume), while Hek is not a substrate for *Mm*PylRS(Y384F). The mutation Y384F was kept in all subsequent PylRS tested. Next, we tested the variant *Mm*PylRS(Y306A/Y384F), a well characterized unspecific synthetase accepting a broad spectrum of different amino acids [18,19,20]. With this variant we were able to incorporate Hek, but Pek incorporation was decreased. To find a variant for efficient Pek incorporation, we identified more residues limiting the size of the amino acid binding pocket. Residues L309 and C348 are often altered in PylRS variants evolved for amino acids with bulky head groups and structural similarity to Pek and Hek [3], but have not been rationally engineered. We introduced the mutations L309A and C348A alone and in combinations. Both allowed incorporation of Pek with good yields, with *Mm*PylRS(C348A/Y384F) being the most efficient variant. The combination of Y306A and L309A reduced the protein yield drastically, the combination of L309A and C348A slightly. All variants tested are show in Figure 2A. These findings indicate that flexible amino acids may accommodate the binding pocket of *Mm*PylRS in different orientations, but an overly large cavity in the binding pocket allows for too much movement, thus reducing the efficiency of the reaction.

EGFP(150Hek) was expressed with *Mm*PylRS(Y306A/Y384F) with a yield of approx. 2 mg per liter of culture, EGFP(150Pek) could be expressed with *Mm*PylRS(L309A/Y384F), *Mm*PylRS(C348A/Y384F) or *Mm*PylRS(L309A/C348A/Y384F) with a maximum yield of approx. 3 mg protein per liter of culture achieved with *Mm*PylRS(C348A/Y384F) after purification (see Figure 2B). The presence of the noncanonical amino acids was confirmed by full-protein mass spectroscopy (Figure 2C). It should be noted, that the purified EGFP(Pek) showed an additional minor peak which probably corresponds to EGFP(150Q). tRNA^Gln^ has some affinity to the amber stop codon and can be incorporated, when other suppression reactions are slow [21]. This can potentially be alleviated by optimization of expression conditions and use of additional noncanonical amino acid.

**Scheme 1:**
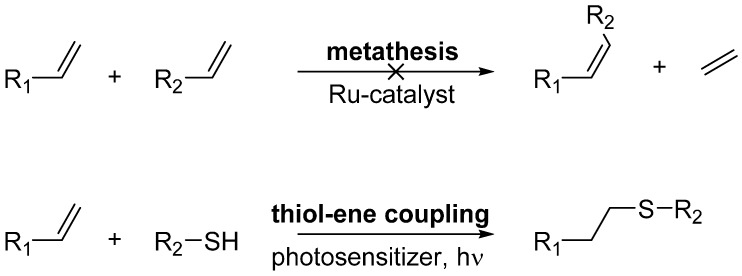
Attempted posttranslational decoration methods. Despite our best efforts, we could not show olefin metathesis with our model proteins.

To assess the reactivity of the terminal double bond, Pek was incorporated into the lipase TTL, which withstands a wide range of medium conditions and constitutes a better model for chemical decorations of enzymes. We expressed TTL(C221Pek) with a TEV protease recognition site upstream of the C-terminal 6xHis tag used for purification, as we speculated that the presence of six histidine residues may interfere with our decoration chemistry (see Scheme 1). As both **1** and **2** readily reacted with allyl alcohol under aqueous conditions (see Supplemental Scheme S1 and Table S3), we first attempted modification of the incorporated amino acids via olefin cross-metathesis. However, despite our best efforts, we were not able to perform olefin cross metathesis following published protocols [10,22], indicating a limitation of the methodology to highly reactive ncAAs with allyl chalcogenide moieties [23] and proteins stable under labeling conditions. In 2008, olefin metathesis on a recombinant protein has been reported by the group of Davis, using the special reactivity of an allylthiol ether installed on subtilisin with 185 equivalents of precatalyst and 9250 equivalents of cross-coupling reagent per equivalent of protein in a buffered solution containing 30% of tBuOH to ensure catalyst solubility [12]. Since this first attempt further investigations by Davis and Schultz did not show significant improvements [10,24]. This indicates that there are many difficulties for aqueous olefin metathesis to be solved.

**Figure 3:**
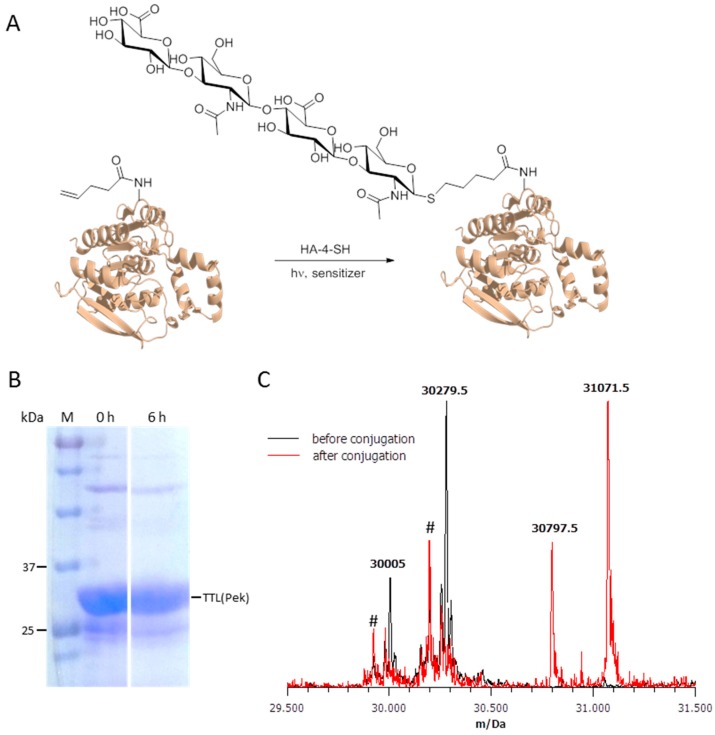
Posttranslational decoration of Thermoanaerobacter thermohydrosulfuricus lipase containing Pek (TTL(221Pek)). (**A**) Schematic representation of the decoration reaction. TTL(221Pek) undergoes light-induced radical conjugation with the thiolic tetrasaccharide HA-4-SH, forming a stable thioether bond; (**B**) SDS-PAGE of purified TTL(Pek) after tag removal with TEV protease before the decoration reaction and after 6 h of reaction time; (**C**) Mass analysis of TTL(Pek) (calculated mass 30,280 Da, found mass 30,279.5 Da) and conjugated TTL(Pek-S-HA-4) (calculated mass 31,071 Da, found mass 31,071.5 Da). For both proteins, an additional peak was found approximately 275 Da below the expected main peak which may be caused by removal of the C-terminal amino acids Gln and Phe due to unspecific TEV protease activity. The additional peaks at app. 30,200 Da and 29,950 Da (marked with #) are caused by TTL(221Gln) present in both preparations due to unspecific stop codon read-through, and the truncated variant resulting from unspecific TEV activity.

Indeed, while olefin metathesis has been applied for protein decoration [25,26], it appears that the metathesis conditions described in those protocols have a detrimental impact on our model proteins. After testing each component alone and in combination with the modified protein, we elucidated that EGFP and TTL show strong precipitation only in the presence of the Hoveyda-Grubbs II catalyst. A possible explanation for this could be the reaction of the ruthenium catalyst with lysine or cysteine side chains leading to precipitation and breakdown of the protein structure. Nevertheless, we could show nearly quantitative thiol-ene coupling of the anomeric thiol tetrasaccharide HA-4-SH (details on HA-4-SH will be available in a forthcoming publication [27]) and the TTL(221Pek). This photochemical reaction proceeds by a radical mechanism to give the corresponding anti-Markovnikov-type thioether. As TTL does not contain cysteines and therefore the thiol cannot be attached via disulfide formation, this coupling reaction demonstrates the feasibility of our chosen ncAAs for specific posttranslational chemical decoration. The results are summarized in Figure 3. It should be noted that in some reactions, a peak was found at a mass 17 Da higher than expected (Figure S1). A possible explanation for this is an oxidation or hydroxylation during the HPLC separation. Additional peaks in the mass spectra are caused by unspecific TAG read-through with Gln, as described above, and additionally by unspecific TEV protease activity, cleaving two additional amino acids. An optimization of the protein expression and TEV cleavage should reduce these unspecific occurrences.

In conclusion, we present the first incorporation of Hek using the well characterized variant *Mm*PylRS(Y306A/Y384F) and the first incorporation of Pek with acceptable yield using novel rational variants, in particular *Mm*PylRS(C348A/Y384F). These amino acids allow posttranslational protein decoration and also for various tether lengths. The amino acid specificity of novel *Mm*PylRS variants should be further assessed, as they are potentially promiscuous, enabling the incorporation of a wide array of novel noncanonical amino acids, also providing a basis for further engineering of *Mm*PylRS.

## 3. Discussion

We used orthogonal protein expression and subsequent chemoselective thiol-ene conjugation of carbohydrates to produce biocompatible protein scaffolds potentially useful for noninvasive targeting in living systems. In particular, the site-specific conjugation of carbohydrate moieties on proteins decorated with olefins as biorthogonal tags offers new tools for the defined targeting and structural and functional investigation of various phenomena in the living cells. In this context, the significance of this work is threefold: We present a method of incorporating long-chain olefins into proteins in a site-specific manner and show the conjugation with a polysaccharide as a model reaction for protein surface decorations which mimics natural glycosylations and other relevant modifications. We speculate that access to protein conjugation chemistry occurring at greater tether lengths above the protein surface will increase the tolerance for the introduced conjugation partners. Our method would thus provide a useful tool to generate proteins with artificial glycosylations or other modifications for medicinal research and novel applications.

In addition, the variant *Mm*PylRS(C348A/Y384F) presented in this study should be assessed for the incorporation of bulky ncAAs not currently accepted by known variants. The increase in binding pocket size could allow the activation of a large array of ncAA structures, and give rise to improved engineering strategies for second-generation PylRS variants, employing *Mm*PylRS(C348A/Y384F) as starting sequence, as has previously been the case with MmPylRS/Y306A/Y384F) [20].

Doubtless, the possibility for *in vivo* production of proteins containing metathesis-competent ncAAs, such as Pek and Hek, is especially attractive as metathesis is an important and versatile method of C-C bond formation that is highly chemoselective and should display a high level of bioorthogonality. Therefore, it is extraordinarily attractive to use the olefin metathesis for the chemical modification of proteins. Unfortunately, we could demonstrate that olefin metathesis is not a generally applicable method for protein decoration. The fact that a lipase known for organic solvent stability [28] precipitates under typical reaction conditions in the presence of catalyst clearly indicates the method’s limitation to a select few proteins. The main challenge of using olefin metathesis for bioorthogonal chemistry is the instability of the catalytically active species in the presence of numerous functional groups from the protein. This problem can be potentially circumvented by performing the olefin metathesis reactions on proteins by using alternative solvents, such as supercritical carbon dioxide which is tolerated by numerous proteins and even living cells [29].

## 4. Materials and Methods

### 4.1. Synthesis of Hek

**Scheme 2:**
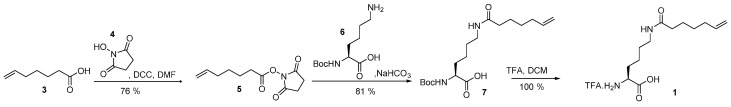
Synthesis of Hek (**1**).

6-Heptenoic acid (**3**) was activated with NHS (**4**) and the activated ester (**5**) subsequently substituted by *N*_α_-Boc protected lysine (**6**). *N*_α_-Boc-*N*_ε_-heptenoyllysine (**7**) was deprotected with TFA to give Hek (**1**) as TFA salt with a good global yield of 61% [30] (see Scheme 2).

### 4.2. Synthesis of Pek

**Scheme 3:**
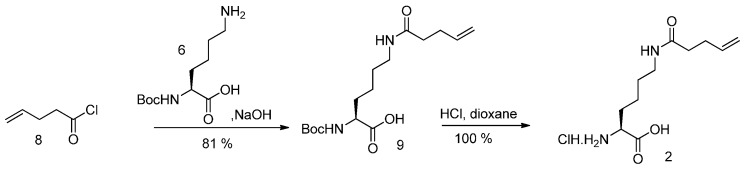
Synthesis of Pek (**2**).

*N*_α_-Boc protected lysine (**6**) reacted with 4-pentenoylchloride (**8**), giving *N*_α_-Boc-*N*_ε_-pentenoyllysine (**9**). Deprotection with HCl gave (**2**) as hydrochloride (see Scheme 3).

### 4.3. Mutagenesis and Expression Tests

Mutations were introduced into *Mm*PylRS cloned to the suppression vector pJZ-P_trp_-*Mm*PylTS [31] with overlapping mutagenic primers following the QuikChange protocol (Agilent, Santa Clara, CA, USA). Variants were cotransformed in *E. coli* BL21(DE3) with pQE80L-EGFP(150amber)-6xHis for expression of the target protein (EGFP) with the mutation N150TAG. Suppression was tested by addition of 2 mM Hek or Pek and 1 mM inducing agent (IPTG) to cultures in mid-log phase (OD_600_ = 0.5–0.7) in shake flasks. Controls without added amino acid, with different amino acids and with wild-type EGFP were prepared in the same way. After expression overnight, samples were taken for expression analysis with western blot and suppressed proteins were purified via immobilized metal affinity chromatography using gravity-flow columns packed with nickel-NTA slurry (IBA, Göttingen, Germany). For storage and mass analysis, the buffer was exchanged to 50 mM Tris-Cl pH 7.5/50 mM NaCl.

For western blot analysis, lysed samples were separated by SDS-PAGE (same amount of lysate applied to each gel pocket), transferred to a PVDF membrane and stained using an antibody directed to the hexahistidin tag of full-length EGFP and a secondary antibody coupled to alkaline phosphatase (all from Abcam, Cambridge, UK) with BCIP/NBT.

Purified proteins were analyzed with SDS-PAGE and HPLC-ESI mass spectrometry using a C5 column (Supelco analytical, Sigma-Aldrich, St. Louis, CA, USA) on an Agilent 1260 for separation and a coupled Agilent QTOF 6530 or Bruker microTOF for mass analysis. Measured mass spectra were deconvoluted using software supplied with the instruments using the maximum entropy algorithm.

For chemical decoration, TTL(221Pek) and TTL(221Hek) were expressed analogously. To prevent interference of 6xHis tag during subsequent decoration reactions, the expression construct contained a TEV protease cleavage site directly upstream of the C-terminal 6xHis tag. For tag removal, the proteins were incubated with 0.1 mg TEV protease per mg of TTL in 150 mM NaCl/50 mM Tris-Cl pH 7.5/2 mM DTT overnight and subsequently applied to a nickel-NTA column to retain uncleaved protein and the 6xHis-tagged TEV protease.

### 4.4. Metathesis Reactions

To 200 µL of EGFP (0.12 mg·mL^−1^ in 0.25 M PBS pH 6) were added MgCl_2_·6H_2_O (10,000 eq 1.5 mg), allylic alcohol (10,000 eq, 0.5 µL ) and 86 µL of a solution of 0.7 mg of HGII in tBuOH (386 µL). To 100 µL of TTL(Pek) (1.5 mg·mL^−1^ in 0.25 M PBS pH 6) were added MgCl_2_·6H_2_O (10,000 eq, 2.9 mg), allylic alcohol (10,000 eq, 1 µL) and 100 µL of a solution of 3.0 mg of HGII in tBuOH (500 µL). To 100 µL of TTL(Hek) (0.2 mg·mL^−1^ in 0.25 M PBS pH 6) were added MgCl_2_·6H_2_O (10,000 eq 1.4 mg), allylic alcohol (0.4 µL, 10,000 eq) and 100 µL of a solution of 0.4 mg of HGII in tBuOH (500 µL). The mixtures were stirred at room temperature and the reaction was followed by mass spectroscopy. No desired product of the cross metathesis reaction was observed and the signal of the protein was no longer observed.

### 4.5. Thiol-Ene Coupling

The thiolated tetrasaccharide HA-4-SH (34 mg, 42.9 µmol, 1000 eq), synthesized in the group of J. Rademann [27], and the photosensitizer Vazzo44 (2.76 mg, 8.6 µmol, 200 eq.) were added to a degassed solution of TTL(221Pek) (1 mL, 1.3 mg/mL in 0.25 M PBS pH 6) in a UV-cuvette. The reaction mixture was irradiated with UV light from an Exo Terra Repti Glo 5.0 Compact UVB100, 25 W No. PT2187 under continuous gentle stirring for 6 h, with addition of further 2.76 mg of Vazzo44 after 4 h. Afterwards the protein solution was dialyzed against 50 mM Tris-Cl pH 7.5/50 mM NaCl.

## Supplementary Materials

Supplementary materials can be accessed at: http://www.mdpi.com/1420-3049/21/3/287/s1.

## Abbreviations

The following abbreviations are used in this manuscript:

ncAA: noncanonical amino acid
*Mm*PylRS: *Methanosarcina mazei* pyrrolysyl-tRNA synthetase
*Mb*PylRS: *Methanosarcina barkeri* pyrrolysyl-tRNA synthetase
EGFP: enhanced green fluorescent protein
TTL: *Thermoanaerobacter thermohydrosulfuricus* lipase
Hek: *N*_ε_-heptenoyl lysine
Pek: *N*_ε_-pentenoyl lysine
HGII: Hoveyda-Grubbs catalyst, second generation
