# Supplementary material for: Incorporation of Amino Acids with Long-Chain Terminal Olefins into Proteins
Source: Molecules. 2016 Feb 29;21(3):287. doi: 10.3390/molecules21030287 (PMC6272937; doi:10.3390/molecules21030287)
Supplement: Supplementary file 1 [file molecules-21-00287-s001.pdf]

Supplementary Materials: Incorporation of Amino Acids with Long-Chain Terminal Olefins into Proteins

Matthias P. Exner, Sebastian Köhling, Julie Rivollier, Sandrine Gosling, Puneet Srivastava, Zheni I. Palyancheva, Piet Herdewijn, Marie-Pierre Heck, Jörg Rademann and Nediljko Budisa

Supplemental Materials and Methods

Plasmid Sequences

Suppression Plasmid

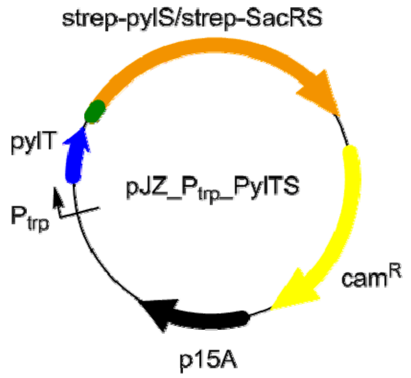

The pJZ plasmid was recently used for efficient incorporation of noncanonical amino acids into proteins [1] using a PylRS variant. The same setup was used for suppression of target genes with SacRS.

Relevant sequence part (pylT-pylS with promoter/terminator):

CGCACCGGTG*cttactccccatcccctgttgacaattaatcatcgaactagttaactagtacgcaggggca*GGTACCCGGGGATCC
GGAAACCTGATCATGTAGATCGAATGGACTCTAAATCCGTTCAGCCGGGTTAGATTCCCGG
GGTTTCCGCCACCTAGGTTTAAACTAAGGAGGTTAATTAA***ATGAGCGCTTGGAGCCACCCGC***
AGTTCGAAAAACTCGAG**ATGGATAAAAAACCACTAAACACTCTGATATCTGCAACCGGG**
CTCTGGATGTCCAGGACCGGAACAATTCATAAAATAAAACACCACGAAGTCTCTCGAA
GCAAAATCTATATTGAAATGGCATGCGGAGACCACCTTGTTGTAAACAACTCCAGGAG
CAGCAGGACTGCAAGAGCGCTCAGGCACCACAAATACAGGAAGACCTGCAAACGCTG
CAGGGTTTCGGATGAGGATCTCAATAAGTTCCTCACAAAGGCAAACGAAGACCAGACA
AGCGTAAAAGTCAAGGTCGTTTCTGCCCCTACCAGAACGAAAAAGGCAATGCCAAAAT
CCGTTGCGAGAGCCCCGAAACCTCTTGAGAATACAGAAGCGGCACAGGCTCAACCTTC
TGGATCTAAATTTTCACCTGCGATACCGGTTTCCACCCAAGAGTCAGTTTCTGTCCCGG
CATCTGTTTCAACATCAATATCAAGCATTTCTACAGGAGCAACTGCATCCGCACTGGTA
AAAGGGAATACGAACCCCATTACATCCATGTCTGCCCCTGTTCAGGCAAGTGCCCCCGC
ACTTACGAAGAGCCAGACTGACAGGCTTGAAGTCCTGTTAAACCCAAAAGATGAGATT
TCCCTGAATTCCGGCAAGCCTTTCAGGGAGCTTGAGTCCGAATTGCTCTCTCGCAGAAA
AAAAGACCTGCAGCAGATCTACGCGGAAGAAAGGGAGAATTATCTGGGGAAACTCGA
GCGTGAAATTACCAGGTTCTTTGTGGACAGGGGTTTTCTGGAAATAAAATCCCCGATCC
TGATCCCTCTTGAGTATATCGAAAGGATGGGCATTGATAATGATACCGAACTTTCAAAA
CAGATCTTCAGGGTTGACAAGAACTTCTGCCTGAGACCCATGCTTGCTCCAAACCTTTA
CAACTACCTGCGCAAGCTTGACAGGGCCCTGCCTGATCCAATAAAAATTTTTGAAATAG
GCCCATGCTACAGAAAAGAGTCCGACGGCAAAGAACACCTCGAAGAGTTTACCATGCT
GAACTTCTGCCAGATGGGATCGGGATGCACACGGGAAAATCTTGAAAGCATAATTACG
GACTTCCTGAACCACCTGGGAATTGATTTCAAGATCGTAGGCGATTCCTGCATGGTCTa
TGGGGATACCCTTGATGTAATGCACGGAGACCTGGAACTTTCCTCTGCAGTAGTCGGA

CCCATACCGCTTGACCGGGAATGGGGTATTGATAAACCCTGGATAGGGGCAGGTTTCG GGCTCGAACGCCTTCTAAAGGTTAAACACGACTTTAAAAATATCAAGAGAGCTGCAAG GTCCGAGTCTTACTATAACGGGATTTCTACCAACCTGTAACTGCAG*tttcaaacgctaaattgcctgatgc gctacgcttatcaggcctacatgatctctgcaatatattgagtttgcgtgcttttgtaggccggataaggcgttcacgccgcatccggcaagaaacagcaaacaa tccaaaacgccgcgttcagcggcgtttttctgcttt*GCGGCCGCAAGCTT

Target Gene Plasmid

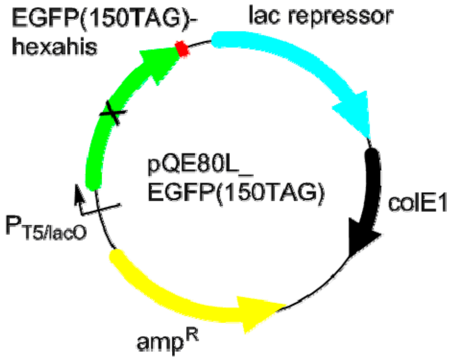

The model target gene was cloned on a commercially available high-copy vector pQE80L (Qiagen, Hilden, Germany) with an additional plasmid-based lac repressor for inducible target protein production in any host strain.

Relevant sequence part (EGFP with promoter/terminator):

CTCGAG*aaatcataaaaaatttatttgctttgtgagcggataacaattataatagattcaattgtgagcggataacaatttcacaca*GAATTCATT AAAGAGGAGAAATTAACT**ATGGTGAGCAAGGGCGAGGAGCTGTTCACCGGGGTGGTG CCCATCCTGGTCGAGCTGGACGGCGACGTAAACGGCCACAAGTTCAGCGTGTCCGGCG AGGGCGAGGGCGATGCCACCTACGGCAAGCTGACCCTGAAGTTCATCTGCACCACCGG CAAGCTGCCCGTGCCCTGGCCCACCCTCGTGACCACCCTGACCTACGGCGTGCAGTGCT TCAGCCGCTACCCCGACCACATGAAGCAGCACGACTTCTTCAAGTCCGCCATGCCCGAA GGCTACGTCCAGGAGCGCACCATCTTCTTCAAGGACGACGGCAACTACAAGACCCGCG CCGAGGTGAAGTTCGAGGGCGACACCCTGGTGAACCGCATCGAGCTGAAGGGCATCG ACTTCAAGGAGGACGGCAACATCCTGGGGCACAAGCTGGAGTACAACTACAACAGCC AC**TAG**GTCTATATCATGGCCGACAAGCAGAAGAACGGCATCAAGGTGAACTTCAAGAT CCGCCACAACATCGAGGACGGCAGCGTGCAGCTCGCCGACCACTACCAGCAGAACACC CCCATCGGCGACGGCCCCGTGCTGCTGCCCGACAACCACTACCTGAGCACCCAGTCCG CCCTGAGCAAAGACCCCAACGAGAAGCGCGATCACATGGTCCTGCTGGAGTTCGTGAC CGCCGCCGGGATCACTCTCGGCATGGACGAGCTGTACAAG*CATCACCATCACCATCAC*TA A**GCTTAATTAGCTGAG*cttggactcctgttgatagatccagtaatgacctcagaactccatctggatttgttcagaacgctcggttgccgcgg gcgtttttattggtgagaatccaag*CTAGCTTGGCG

Strain Genotypes

Table S1. Bacterial strains used in this study.

Name	Genotype	Source
BL21(DE3)	*fhuA2 [lon] ompT gal (λ DE3) [dcm] ΔhsdS*	NEB, Ipswich, USA

Primer Sequences

Table S2. Primers used in this study.

Name	Sequence	Used for
MmPylRS_Y306A_for	GCTTGCTCCAAACCTTGCCAACTACCTGCGCAAG	mutagenesis Y306A of *Mm*PylRS(Y384F)
MmPylRS_Y306A_rev	CTTGCGCAGGTAGTTGGCAAGGTTTGGAGCAAGC	mutagenesis Y306A of *Mm*PylRS(Y384F)
PylRS_L309A_for	CAAACCTTGCCAACTACGCGCGCAAGCTTGACAGG	mutagenesis L309A of *Mm*PylRS(Y384F)
PylRS_L309A_rev	CCTGTCAAGCTTGCGCGCGTAGTTGGCAAGGTTTG	mutagenesis L309A of *Mm*PylRS(Y384F)
MmPylRS_L309AvY306A_for	CAAACCTTGCCAACTACGCGCGCAAGCTTGACAGG	mutagenesis L309A of *Mm*PylRS(Y306A/Y384F)
MmPylRS_L309AvY306A_rev	CCTGTCAAGCTTGCGCGCGTAGTTGTAAAGGTTTG	mutagenesis L309A of *Mm*PylRS(Y306AY384F)
PylRS_C348A_for	CATGCTGAACTTCGCCCAGATGGGATCGG	mutagenesis C348A of *Mm*PylRS(Y384F) or *Mm*PylRS(L309A/Y384F)
PylRS_C348A_rev	CCGATCCCATCTGGGCGAAGTTCAGCATG	mutagenesis C348A of *Mm*PylRS(Y384F) or *Mm*PylRS(L309A/Y384F)

Detailed Syntheses

All reagents are commercial grade (purchased from Sigma-Aldrich) and were used as received. All reactions were performed under inert atmosphere using anhydrous solvents which were dried and distilled before use. Thin-layer chromatography (TLC) and flash chromatography separations were respectively performed on precoated silica gel 60 F 254 plates (Merck, 0.25 mm) and on Merck silica gel 60 (230–400 mesh). ^{1}H-NMR spectra were recorded at 400 MHz; shifts are relative to internal TMS. ^{13}C-NMR spectra were recorded at 100 MHz.

Synthesis of Hek (1)

2,5-Dioxopyrrolidin-1-yl hept-6-enoate (**5**)

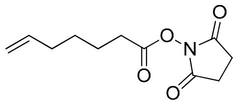

A mixture of 6-heptenoic acid **3** (0.53 g, 4.13 mmol, 1 eq), *N*-hydroxysuccinimide (0.47 g, 4.13 mmol, 1 eq) and DCC (0.85 g, 4.13 mmol, 1 eq) was stirred at room temperature for 48 h. The resulting white precipitate was filtered and washed with DMF. The filtrate was diluted with AcOEt and washed with water. The aqueous phase was extracted with AcOEt. The organic layers were combined, dried over Na_2SO_4, filtered and concentrated under reduced pressure. The residue was purified on silica gel flash chromatography (Heptane/AcOEt, 70:30) to give 2,5-dioxopyrrolidin-1-yl hept-6-enoate **5** as a white oil (0.707 g, 76%).

^{1}H-NMR (400 MHz, $CDCl_3$, δ in ppm) δ: 5.83–5.74 (m, 1H, C**H**=CH_2), 5.05–4.96 (m, 2H, CH=C**H**$_2$), 2.83 (s, 4H, NCOC**H**$_2$), 2.60 (t, *J* = 8.0 Hz, 2H, COC**H**$_2$$CH_2$), 2.13–2.07 (m, 2H, CH_2C**H**$_2$CH), 1.76 (q, *J* = 7.6 Hz, 2H, CH_2C**H**$_2$$CH_2$$CH_2$), 1.51 (q, *J* = 7.6 Hz, 2H, $CH_2$$CH_2$C**H**$_2$$CH_2$). ^{13}C-NMR (100 MHz, $CDCl_3$,

δ in ppm) δ: 170.5 (2C, N**C**O), 169.3 (O**C**O), 138.6 (**C**H=CH$_2$), 115.2 (CH=**C**H$_2$), 36.0 (**C**H$_2$CH=CH$_2$), 30.5 (OCO**C**H$_2$), 27.6 (CH$_2$CH$_2$**C**H$_2$CH$_2$), 25.8 (2C, CO**C**H$_2$**C**H$_2$CO), 24.2 (CH$_2$**C**H$_2$CH$_2$CH$_2$).

(S)-2-Boc-amino-6-(hept-6-enamido)hexanoic acid (**7**)

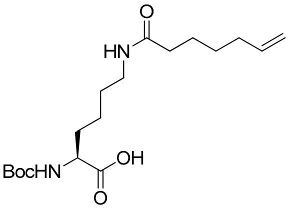

A mixture of 2,5-dioxopyrrolidin-1-yl hept-6-enoate **5** (0.718 g, 3.18 mmol, 1 eq), Boc-Lysine **6** (0.778 g, 3.18 mmol, 1 eq) in aqueous $NaHCO_3$ (1 N, 20 mL) was stirred at room temperature for 20 h and then acidified to pH 2 by adding 1 N HCl. The reaction mixture was then extracted with AcOEt. The combined organic layers were washed with HCl 1 N, H_2O, dried over Na_2SO_4, filtered and concentrated under reduce pressure to give (*S*)-2-Boc-amino-6-(hept-6-enamido)hexanoic acid **7** as a colorless oil (1.08 g, 95%).

^{1}H-NMR (400 MHz, $CDCl_3$, δ in ppm) δ: 5.86–5.76 (m, 1H, C**H**=CH$_2$), 5.79 (s broad, 1H, N**H** amide), 5.30 (s broad, 1H, N**H**Boc), 5.04–4.96 (m, 2H, CH=C**H**$_2$), 4.29 (s, 1H, BocNHC**H**), 3.30–3.26 (m, 2H, CH$_2$C**H**$_2$NH), 2.20 (t, *J* = 7.6 Hz, 2H, COC**H**$_2$CH$_2$), 2.09–2.07 (m, 2H, CH$_2$C**H**$_2$CH), 1.77–1.53 (m, 10H, BocNHCHC**H**$_2$C**H**$_2$C**H**$_2$CH$_2$ + COCH$_2$C**H**$_2$C**H**$_2$CH$_2$CH), 1.47 (s, 9H, Boc). ^{13}C-NMR (100 MHz, $CDCl_3$, δ in ppm) δ: 175.3 (NH**C**O), 174.0 (**C**OOH), 157.9 (**C**O Boc), 138.3 (**C**H=CH$_2$), 114.6 (CH=**C**H$_2$), 79.8 (Cq, Boc), 49.3 (BocNH**CH**), 39.2 (**C**H$_2$NH), 36.4 (CO**C**H$_2$), 33.3 (CH$_2$CH$_2$CHCH$_2$), 30.8 (BocNHCH**C**H$_2$), 28.4 (**C**H$_2$CH$_2$NH), 28.3 (**C**H$_2$CH$_2$CHCH$_2$), 28.3 (3 × CH$_3$ Boc), 24.8 (COCH$_2$**C**H$_2$), 22.4 (BocNHCHCH$_2$**C**H$_2$). HRMS (ESI+): calculated for $C_{18}H_{33}N_2O_5$ $[M + H]^+$ 357.4705, found 357.4711.

(S)-2-amino-6-(hept-6-enamido)hexanoic acid, TFA salt (**1**)

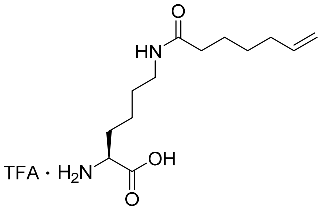

To a solution of (*S*)-2-Boc-amino-6-(hept-6-enamido)hexanoic acid **7** (1.08 g, 3.03 mmol, 1 eq) in DCM, was added TFA (2 mL). The solution was stirred 4 h at room temperature and concentrated. The residue was dissolved in water, washed with AcOEt and concentrated to give (*S*)-2-amino-6-(hept-6-enamido)hexanoic acid, TFA salt **1** as a white solid (0.707 g, 64%).

^{1}H-NMR (400 MHz, D_2O, δ in ppm) δ: 5.92–5.82 (m, 1H, C**H**=CH$_2$), 5.06–4.96 (m, 2H, CH=C**H**$_2$), 3.97 (t, *J* = 6.4 Hz, 1H, NH$_2$C**H**COOH), 3.18 (t, *J* = 6.8 Hz, 2H, CH$_2$C**H**$_2$NH), 2.22 (t, *J* = 7.6 Hz, 2H, COC**H**$_2$CH$_2$), 2.07–1.83 (m, 4H, CH$_2$C**H**$_2$CH + NH$_2$CHC**H**$_2$CH$_2$CH$_2$CH$_2$), 1.61–1.33 (m, 8H, NH$_2$CHCH$_2$C**H**$_2$C**H**$_2$CH$_2$ + COCH$_2$C**H**$_2$C**H**$_2$CH$_2$CH). ^{13}C-NMR (100 MHz, D_2O, δ in ppm) δ: 177.0 (NH**C**O), 174.6 (**C**OOH), 139.5 (**C**H=CH$_2$), 114.4 (CH=**C**H$_2$), 54.5 (NH$_2$**C**HCOOH), 38.8 (**C**H$_2$NH), 36.3 (CO**C**H$_2$), 32.6 (CH$_2$**C**H$_2$CHCH$_2$), 30.0 (NH$_2$CH**C**H$_2$), 28.0 (**C**H$_2$CH$_2$NH), 27.4 (**C**H$_2$CH$_2$CHCH$_2$), 24.9(COCH$_2$**C**H$_2$), 21.8 (NH$_2$CHCH$_2$**C**H$_2$). HRMS (ESI+): calculated for $C_{13}H_{25}N_2O_3$ $[M + H]^+$ 257.1860, found 257.1855.

Synthesis of Pek (**2**)

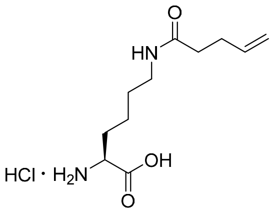

Commercially available *N*-*α*-Boc-L-lysine **6** (2.5 g, 10 mmol, 1 eq) was dissolved in 1 N NaOH (50 mL) solution in dioxane (1:1) and cooled in an ice bath. To this pre-cooled solution was added commercially available pentenoyl chloride **8** (2.3 mL, 21.0 mmol, 2.1 eq) dropwise and the solution left to stir overnight (0 °C to r.t.). After the completion of the reaction as indicated by a TLC analysis, the solution was cooled again and acidified with 1 N aq. HCl and extracted with dichloromethane. The organic extracts were combined, dried over Na_2SO_4, evaporated and chromatographed to obtain the $N_ε$-pentenoyl product **9** in quantitative yield. This product was again dissolved in dry dioxane (10 mL) and cooled to 0 C. 4 N HCl in dioxane (10 mL) was then added to this solution drop-wise and the reaction left to stir at r.t. overnight. The solvent was then evaporated and the yellowish residue dissolved in water and washed with diethyl ether. The aqueous layer was then lyophilized and purified on reverse phase HPLC (C-18; H_2O:CH_3CN) to obtain the product $N_ε$-pentenoyllysine hydrochloride salt **2** as white powder.

^{1}H-NMR (400 MHz, D_2O, δ in ppm): δ 5.88-5.79 (m, 1H, C**H**=CH_2), 5.10–5.01 (m, 2H, CH=C**H**$_2$), 4.03 (t, *J* = 6 Hz, 1H, NH_2C**H**COOH), 3.18 (t, *J* = 7 Hz, 2H, CH_2C**H**$_2$NH), 2.32 (m, 4H, COC**H**$_2$$CH_2$ + CH_2C**H**$_2$CH), 1.99–1.89 (m, 2H, NH_2CHC**H**$_2$$CH_2$$CH_2$$CH_2$), 1.57–1.49 (m, 2H, NH_2CHCH$_2$C**H**$_2$$CH_2$$CH_2$), 1.47–1.27 (m, 2H, NH_2CHCH$_2$$CH_2$C**H**$_2$$CH_2$); ^{13}C-NMR (75 MHz, D_2O): δ 175.7 (NH**C**O), 172.0 (**C**OOH), 136.7 (**C**H=CH_2), 115.3 (CH=**C**H$_2$), 52.6 (NH_2**C**HCOOH), 38.3 (**C**H$_2$NH), 34.7 (CO**C**H$_2$), 29.1 (CH_2**C**H$_2$CHCH$_2$), 27.5 (NH_2CH**C**H$_2$$CH_2$), 24.8 (**C**H$_2$$CH_2$NH), 21.2 ($NH_2$CHCH$_2$**C**H$_2$) ; HRMS (ESI+): calculated for $C_{11}H_{21}N_2O_3$ $[M + H]^+$ 229.1546, found 229.1549.

Synthesis of HA-4-SH

A detailed synthesis of HA-4-SH will be available elsewhere.

Aqueous Metathesis with Protected 1 and 2

Cross Metathesis of NBoc-methylester pentenoyllysine (*n* = 1) as well as NBoc-methylester heptenoyllysine (*n* = 3) has been studied with various olefin partners in the presence of Hoveyda Grubbs II in aqueous media or organic conditions. Our full study (various experimental conditions and CM partners) will be reported elsewhere.

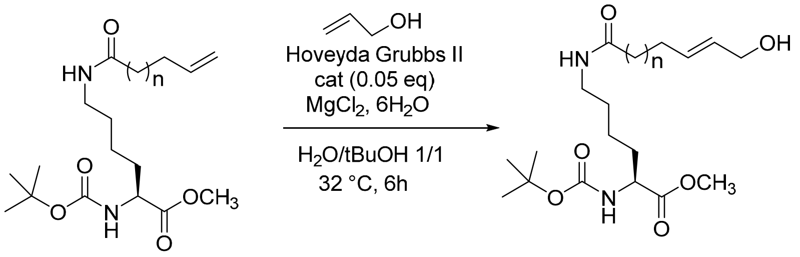

Scheme S1. Aqueous metathesis with protected **1** and **2** with allyl alcohol as coupling partner.

The metathesis results with allyl alcohol as coupling partner are summarized in

Table S3. Results of cross metathesis in aqueous and organic solvent systems.

Entry	*n*	Solvent	Additive $MgCl_2$	Yield of CM Product
1	*n* = 1	CH_2Cl_2	no	<10%
2	*n* = 1	H_2O/*t*BuOH 1/1	1.5 eq	55%
3	*n* = 3	CH_2Cl_2	no	40%
4	*n* =3	H_2O/*t*BuOH 1/1	1.5 eq	60%–70%

Yields of CM and reactions were improved up in aqueous media (H_2O/tBuOH 1/1) with Hoveyda Grubbs II catalyst (0.05 eq–0.1 eq), allyl alcohol (10 eq) and addition of $MgCl_2$ (1.5 eq) at 32 °C for 2 to 6 h. Surprisingly the yields were inferior when the reactions were run in CH_2Cl_2 (approximately 10%–40% compared to 55%–60% isolated yield in aqueous media), showing the need of $MgCl_2$ to prevent non-productive chelation.

Supplemental Figures

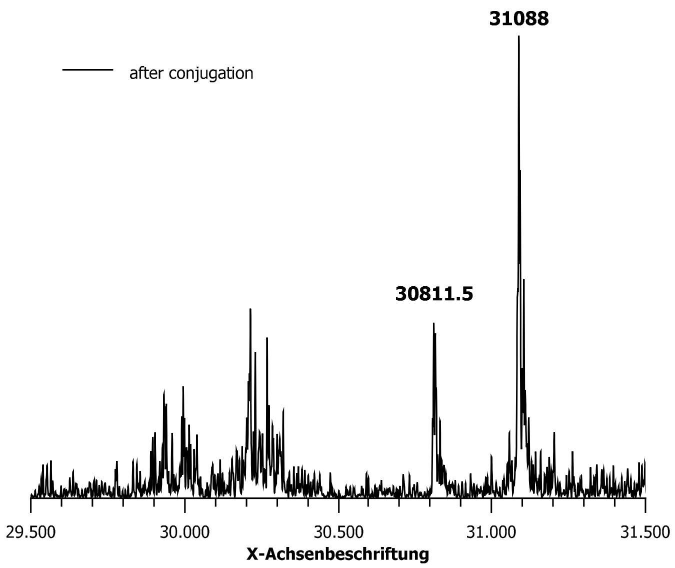

Figure S1. Mass analysis of TTL(221Pek) after 6xHis tag removal conjugated with HA-4-SH.

The expected mass is 31071 Da, the difference of 17 Da can be explained by a hydroxylation or oxidation during the analysis. The lower-mass peak is caused by unspecific activity of the TEV protease used to remove the 6xHis tag prior to the decoration reaction.

Supplemental References

1. Al Toma, R.S.; Kuthning, A.; Exner, M.P.; Denisiuk, A.; Ziegler, J.; Budisa, N.; Süssmuth, R.D. Site-Directed and Global Incorporation of Orthogonal and Isostructural Noncanonical Amino Acids into the Ribosomal Lasso Peptide Capistruin. *ChemBioChem* **2015**, *16*, 503–509.
